# Personalized Medicine in Oncology; a Special Issue of the *Journal of Personalized Medicine*

**DOI:** 10.3390/jpm11070632

**Published:** 2021-07-02

**Authors:** Ari VanderWalde

**Affiliations:** West Cancer Center and Research Institute, Memphis, TN 38138, USA; avanderw@westclinic.com

Nowhere is the explosion in comprehensive genomic testing more evident than in oncology. Multiple consensus guidelines now recommend molecular testing as part of the standard of care for most metastatic tumors. To aid in the advancement of this rapidly changing field, we intended this Special Issue of *JPM* to focus on technical developments in the genomic profiling of cancer, detail promising somatic alterations that either are, or have a high likelihood of being, relevant in the near future, and to address issues related to the pricing and value of these tests.

The last few years have seen the cost of molecular testing decrease by orders of magnitude. In 2018, we saw the first “site-agnostic” drug approvals in cancer (for microsatellite unstable cancer (PD-1 inhibitors) and NTRK-fusions (TRK inhibitors)). This has recently been followed by pan-tumor approvals for tumors that have a high tumor mutational burden. Research on targetable mutations, determination of genetic “signatures” that can use multiple individual genes/pathways, development of targeted therapy, and insight into the value of new technology remains at the cutting edge of research in this field. In this Special Issue of *JPM* we solicited papers that present new technologies to assess predictive biomarkers in cancer, conducted original research (pre-clinical or clinical) that demonstrates promise for particular targeted therapies in cancer, and articles that explore the clinical and financial impacts of this paradigmatic shift in cancer diagnostics and treatment.

In this issue, four review articles and a commentary present great depth in biomarker testing both in the clinic as well as in early science.

Two papers discuss the role of tumor biomarkers in the diagnosis and treatment of various malignancies. Stein et al. present a comprehensive review of the molecular and immunologic biomarkers that have led to approvals in more than one malignancy [1]. With a focus on US-FDA approvals as well as studies leading to those approvals, the paper serves as an overview of patient-directed therapy based upon these markers. This paper also performs a deep dive into molecular and genomic biomarkers specifically in metastatic non-small-cell lung cancer; a cancer for which over 50% of cases have a targetable molecular alteration. Miron et al. [2] perform a deep dive into renal cell cancer—another malignancy with genomic and molecular alterations that predispose to response in for both immune therapy and targeted therapy. The authors particularly discuss the development and discovery of these markers and how they are used in a real-world setting.

In addition to these clinical reviews, Cortesi and colleagues provide commentary on the role of on relatively uncommon germline mutations that effect homologous recombination repair (HRR), such as *PALB2*, *CHEK2*, *ATM*, and others [3]. The authors propose that the presence of homologous repair deficiency can often serve as a positive prognostic factor, but that the prediction of whether tumors associated with these genes respond to PARP inhibitor therapy depends on the gene and on the penetrance. The authors assert that tests that can reveal the presence of all HRR genes should be part of a standard germline panel, rather than just focusing on BRCA1 and 2.

In the basic-science realm, Dr. Tachiro Goto presents a review paper describing the role of patient-derived xenografts (PDX) in precision oncology [4]. The paper describes the preparation of PDX and the grafting of human tumor tissue into nude mice as well as much of the current advancement of the field using this model. Due to the preservation of tumor heterogeneity as well as the tumor microenvironment, the paper predicts the use of these relatively novel pre-clinical approaches to biomarker driven testing and therapeutics.

Finally, Dr. Loredana Marcu proposes a different type of biomarker useful in diagnosis and prediction of response or progression in lung cancer [5]. By focusing on the development of imaging-based radiologic biomarkers, the paper paves the way for personalized oncology focused not only on tumor-based genomic alterations, but on the ability of advanced imaging to discover the presence of pathological findings such as the presence of cancer-stem cells, apoptosis, and circulating tumor cells as well as the ability to determine proliferation kinetics.

With the context provided by these reviews and commentaries, intriguing original research is presented in this Special Issue that explores the role of novel biomarkers in specific cancer types, explores demographic “biomarkers” as keys to response to therapy, and details results of operational tests and processes that serve to integrate precision oncology into the clinic.

Two of the original research pieces look specifically at novel biomarkers in gastrointestinal cancers. Rios-Arrabal and colleagues present the role of heme oxygenase-1 (HO-1) as a marker of “stemness” in colorectal cancer [6]. The authors found that HO-1 overexpression is commonly co-expressed with endothelin converting enzyme-1, and that resistance was unrelated to the presence or absence of p53 (a poor prognosis tumor marker). Further exploration led to the conclusion that HO-1 based-therapies could be developed and preferentially used in patients with expression of these markers.

Branchi et al. explored tissue samples obtained from 27 patients with biliary tract adenocarcinomas and assessed them for the presence and density of tumor infiltrating lymphocytes (TIL) [7]. It was found that those cancers with high TIL density exhibited an overall survival almost twice as long as those with low density of TIL. As such, they propose that TIL density could serve as a potential clinical biomarker for prognosis in biliary tract cancer and suggest that this difference shows that there is a key regulator role in the immune landscape in this cancer type.

In ovarian cancer, Ulm and colleagues explore the potential role of integrin associated kinase as a biomarker in this malignancy [8]. Ovarian cancer tissue samples were pair-matched to normal adjacent ovarian tissue from 24 patients and tissue microarray was used to compare gene expression profiles which then led to the identification of molecular pathways for further analysis. Integrin-linked kinase (ILK) emerged as a commonly upregulated pathway in ovarian cancer, and it was shown to be a driver of malignancy. An ILK-1 chemical inhibitor had positive results against ILK-1 expressing tumors in xenograft models. These may serve as initial findings to justify the development of ILK-1 directed therapies in this malignancy.

While precision medicine is usually targeting molecular alterations, it is important to recognize that virtually any clinical, laboratory, or patient-reported factors may potentially serve as markers predicting for various outcomes in cancer. In this issue, Kim et al. present a retrospective study of 97 patients who received the chemotherapeutic regimen FOLFIRINOX for pancreatic cancer [9]. The authors demonstrated better outcomes for women than for men on this regimen, including a trend for improved progression-free-survival and significantly better overall survival, despite a higher median age of women as compared to men. There are of course limitations on the interpretability of this study, but it is an important step to use real-world data to identify not only molecular markers, but demographic markers as well to guide therapy in cancer.

Just as identification of biomarkers in the lab and in clinical trials is important in precision oncology, the integration of these findings into clinical practice is equally, if not more, essential. Taghizadeh and colleagues describe how their real-world precision medicine platform MONDTI (molecular oncologic diagnostics and therapy) at the Medical University of Vienna was able to identify patients with actionable molecular alterations [10]. The group used standard multi-gene next generation sequencing panels and immunohistochemistry and utilized a multidisciplinary team meeting held every other week that made recommendations for off-label use based on levels of evidence. In this retrospective study the authors evaluated what demographic or disease state characteristics were more or less likely to result in recommendations for targeted therapy among the almost 600 patients who went through the MONDTI system. They found that certain types of malignancies were more likely to result in recommendations, that certain genomic alterations were more likely to result in recommendations, and that male gender was more likely to result in recommendations.

A similar article by me and my colleagues described the implementation of a molecular tumor board in a community oncology setting [11]. Rather than focusing on characteristics associated with recommendations, we focused on the types of recommendations made and whether or not the recommendations were followed. Among 613 patients reviewed by the bi-weekly molecular tumor board, 37% of patients had standard therapy recommended, 31% had a recommendation to go onto a clinical trial, germline testing was recommended in 17% of patients, and off-label therapy was recommended in only 10%. Follow-through with recommendations depended on the type of recommendation. Only 13% of those for whom a trial was recommended were able to go onto a study. Standard therapy recommendations were followed almost 80% of the time, while germline testing recommendations were only followed in about a third of patients.

The opportunity to dedicate an entire issue to precision oncology is important. The articles in this issue represent the breadth of what is being done regarding discovery, development, measurement, and integration of precision oncology in patients. While the field has truly blossomed in the last decade, significant work remains to be done. Each of the articles in this Special Issue captures an important piece of the genomic and personalized oncology puzzle. Though some of the puzzle has been solved, what remains will provide us with mysteries to solve for years to come.

## References

[1] Stein M.K., Oluoha O., Patel K., VanderWalde A. (2021). Precision Medicine in Oncology: A Review of Multi-Tumor Actionable Molecular Targets with an Emphasis on Non-Small Cell Lung Cancer. J. Pers. Med..

[2] Miron B., Xu D., Zibelman M. (2020). Biomarker Development for Metastatic Renal Cell Carcinoma: Omics, Antigens, T-cels, and Beyond. J. Pers. Med..

[3] Cortesi L., Piombino C., Toss A. (2021). Germline Mutations in Other Homologous recombination Repair-Related Genes Than *BRCA1/2*: Predictive or Prognostic Factors?. J. Pers. Med..

[4] Goto T. (2020). Patient-Derived Tumor Xenograft Models: Toward the Establishment of Precision Cancer Medicine. J. Pers. Med..

[5] Marcu L.G. (2020). Imaging Biomarkers of Tumour Proliferation and Invasion for Personalised Lung Cancer Therapy. J. Pers. Oncol..

[6] Rios-Arrabal S., Puentes-Pardo J.D., Moreno-SanJuan S., Szuba A., Casado J., Garcia-Costela M., Escudero-Feliu J., Verbeni M., Cano C., Gonzalez-Puga C. (2021). Endothelin-1 as a Mediator of heme Oxygenase-1-Induced Stemness in Colorectal Cancer: Influence of p53. J. Pers. Med..

[7] Branchi V., Jurgensen B., Esser L., Gonzalez-Carmona M., Weismuller T.J., Strassburg C.P., Henn J., Semaan A., Lingohr P., Manekeller S. (2021). Tumor Infiltrating Neutrophils Are Frequently Found in Adenocarcinomas of the Biliary Tract and Their Precursor Lesions with Possible Impact on Prognosis. J. Pers. Med..

[8] Ulm M.A., Redfern T.M., Wilson B.R., Ponnusamy S., Asemota S., Blackburn P.W., Wang Y., ElNaggar A.C., Narayanan R. (2020). Integrin-Linked Kinase Is a Novel Therapeutic Target in Ovarian Cancer. J. Pers. Med..

[9] Kim J., Ji E., Jung K., Jung I.H., Park J., Lee J.-C., Kim J.W., Hwang J.-H., Kim J. (2021). Gender Differences in Patients with Metastatic Pancreatic Cancer Who Received FOLFIRINOX. J. Pers. Med..

[10] Taghizadeh H., Unseld M., Spalt M., Mader R.M., Mullauer L., Fuereder T., Raderer M., Sibilia M., Hoda M.A., Aust S. (2020). Targeted Therapy Recommendations for Therapy Refractory Solid Tumors—Data from the Real-World Precision Medicine Platform MONDTI. J. Pers. Med..

[11] VanderWalde A., Grothey A., Vaena D., Vidal G., ElNaggar A., Bufalino G., Schwartzberg L. (2020). Establishment of a Molecular Tumor Board (MTB) and Uptake of Recommendations in a Community Setting. J. Pers. Med..

